# Imatinib use for gastrointestinal stromal tumors among older patients in Japan and Taiwan

**DOI:** 10.1038/s41598-022-27092-z

**Published:** 2022-12-28

**Authors:** Yuichi Ichinose, Yi-Hsin Yang, Hui-Jen Tsai, Ru-Yu Huang, Takahiro Higashi, Toshirou Nishida, Li-Tzong Chen

**Affiliations:** 1 Division of Health Services Research, Institute for Cancer Control, National Cancer Center, 5-1-1, Tsukiji, Chuo-ku, Tokyo 104-0045 Japan; 2 National Institute of Cancer Research, National Health Research Institutes, No. 367, Sheng-Li Rd., North District, Tainan, 70456 Taiwan; 3 Department of Public Health and Health Policy, School of Medicine, The University of Tokyo, 7-3-1, Hongo, Bunkyo-ku, Tokyo 113-0033 Japan; 4 National Cancer Center Hospital, 5-1-1, Tsukiji, Chuo-ku, Tokyo 104-0045 Japan; 5 Department of Surgery, Japan Community Health Care Organization Osaka Hospital, 4-2-78, Fukushima, Fukushima-ku, Osaka 553-0003 Japan; 6 Department of Internal Medicine, Kaohsiung Medical University Hospital, Kaohsiung Medical University, No. 100, Tzyou 1st Rd., Sanmin Dist, Kaohsiung City, 80756 Taiwan; 7 Department of Oncology, National Cheng Kung University Hospital, National Cheng Kung University, No. 138, Sheng Li Rd., North Dis., Tainan, 70403 Taiwan

**Keywords:** Cancer, Cancer epidemiology

## Abstract

Tyrosine kinase inhibitors (TKIs) improve the prognosis of patients with gastrointestinal stromal tumors (GISTs). We conducted a retrospective cohort study using cancer registries linked with health utilization data in Japan and Taiwan to assess TKI usage in older and non-older patients. Patients diagnosed with GIST (2012–2014) were categorized into the following: adjuvant and advanced/metastatic settings. The duration and patterns of imatinib therapy were compared between the older (aged ≥ 75 years) and non-older (< 75 years) groups. We included 232 Japanese and 492 Taiwanese patients in the adjuvant setting, and 235 Japanese and 401 Taiwanese patients in the advanced/metastatic setting. Older patients had higher proportions of starting with lower doses (< 400 mg/day) than the non-older patients (adjuvant: 22.5% vs. 4.3% [Japan]; 22.5% vs. 10.9% [Taiwan]; advanced/metastatic: 29.6% vs. 7.2% [Japan]; 32.6% vs. 8.1% [Taiwan]; all *p* < 0.01). The median time to stop imatinib was shorter in the older than in the non-older patients (adjuvant: 301 vs. 975 days [Japan], 366 vs. 1028 days [Taiwan]; advanced/metastatic: 423 vs. 542 days [Japan]; 366.5 vs. 837 days [Taiwan]). More older patients with GIST tended to have TKIs at a lower initial dose and a shorter imatinib duration than the non-older patients.

## Introduction

Gastrointestinal stromal tumors (GISTs) are the most common sarcomas in the gastrointestinal tract. They have an incidence of approximately 10–15 per million people in the world, with 10 and 19.7 per million people in Japan and Taiwan, respectively^1–3^. Most GISTs have activating *KIT* or *PDGFRA* mutations that result in uncontrolled cell growth. Tyrosine kinase inhibitors (TKIs), such as imatinib and sunitinib, inhibit the abnormal proliferation of GIST cells^4^. For localized GISTs, adjuvant imatinib therapy is standard for patients with high-risk GISTs after complete resection. TKIs are the mainstay of treatment for advanced/metastatic GISTs; they improve both progression-free survival (PFS) and overall survival (OS) of patients with GISTs^5,6^.

Although the Asian consensus guideline recommends adjuvant therapy with imatinib for 3 years, some clinical trials have reported that imatinib was discontinued in approximately 25% of study participants (25.8% and 25.2% for 1-year and 2-year adjuvant therapy in the EORTC study, respectively; 12.6% and 25.8% for 1-year and 3-year adjuvant therapy in the SSG XVIII/AIO trial, respectively)^7–10^. Similarly, previous research has shown that 18–48% of patients with advanced/metastatic GISTs discontinue treatment prior to disease progression; however, the results were based on single-center studies or small sample sizes^11,12^. Continued TKI use is the key to improving the prognosis of patients with GISTs; however, there is a paucity of data on the long-term usage of TKIs among Asian patients with GISTs.

The median age of patients diagnosed with GISTs is mid-60 s years, and approximately 30% of patients with GISTs are diagnosed at ≥ 70 years^3^. Compared to younger patients, older patients have more comorbidities and physiological decline. Moreover, their treatment type and doses should be selected carefully due to their higher susceptibility to treatment-related complications or toxicities^13^. Therefore, a substantial proportion of older patients with GISTs may not receive standard treatments, and as a result, have poorer outcomes than younger patients: shorter disease-free survival and overall survival. A previous study found that older patients with GISTs underwent adjuvant therapy less frequently and received lower doses than non-older patients^11,14^. Most patients in the clinical trials were < 65 years, and real-world data on older patients are scarce^6,7,15^. Therefore, it is essential to explore the optimal treatment strategy for older patients.

Utilization of both national cancer registries and health insurance claims data makes it possible to identify the clinical practice pattern in the population. In this study, we evaluated the real-world use of imatinib for patients with advanced/metastatic GISTs and patients receiving adjuvant therapy using nationwide data from Japan and Taiwan. We also compared characteristics among the older and non-older populations with GIST in both countries.

## Methods

### Study overview

This was an retrospective cohort study using cancer registries linked with health utilization data. Hospital-Based Cancer Registry (hereafter Japanese Cancer Registry [JCR] for convenience) and Diagnosis Procedure Combination (DPC) survey data were used to construct the Japanese cohort. The Taiwan Cancer Registry (TCR), Taiwan Death Registry, and National Health Insurance (NHI) data were used to construct the Taiwanese cohort. We examined the patterns of TKI use in patients with advanced/metastatic tumors and patients receiving adjuvant therapy after surgery. This was further compared between the older and non-older patients in each patient group.

This research was conducted according to the guidelines of the 1964 Declaration of Helsinki and its later amendments. The study was approved by the Research Ethics Committee of National Health Research Institutes (EC109010D4-E) and National Cancer Center Institutional Review Board (2017-379). By the regulation of data custodians, the analysis of Japanese data was performed in the Division of Health Services Research at the National Cancer Center. Based on the recommendations from the Ministry of Health, Labour, and Welfare regarding confidentiality, only patient groups above 10 were reported in the result. The data analysis in Taiwan was performed in the Kaohsiung Medical University Center, Health and Welfare Data Science Center (HWDC), and Ministry of Health and Welfare. The results from the Taiwanese cohort were reviewed by the HWDC officials to ensure no violation of confidentiality. Only statistics computed from a cell size of ≥ 3 were allowed to be shared. This study was reported using the Strengthening the Reporting of Observational Studies in Epidemiology (STROBE) guidelines for cohort studies^16^.

### Data source in Japan

The JCR is a national database in which all designated and non-designated cancer care hospitals in Japan must input annually the data of newly diagnosed patients with cancer. The JCR covers > 70% of all patients with cancer in Japan^17^. It has information on patients’ demographics, tumor status expressed in the national coding system language, and the first treatment course. In this study, we identified patients diagnosed with GIST between January 2012 and December 2014 using the JCR data.

The DPC survey data is insurance claims-like data mainly used for medical payments in Japan. The DPC survey data includes patients’ demographics, diagnoses, surgeries, medication prescriptions, admission and discharge status, and other data. The DPC data were collected on a routine basis to monitor the quality/patterns of cancer care up to the end of the following year from diagnosis^18,19^. Given the longer follow-up period in this study, we solicited the hospitals to provide follow-up data up to 2018. Since the longer follow-up data did not have the link key to the JCR, we merged these data using the patient’s sex, year and month of birth, and the date and claims code of GIST-related treatments. Patients with data in only one dataset or patients with no matching counterpart were excluded.

### Data source in Taiwan

The TCR is a national database in which all new cancer cases diagnosed in hospitals with > 50 beds in Taiwan are registered. The TCR covers > 97% of cancer cases in Taiwan^20^. The TCR has two cancer registration types: the long-form database for leading cancers (cancers of the oral cavity and pharynx, colon and rectum, liver, lung, breast, and cervix uteri) and the short-form database for other cancer sites. The long-form database includes more details on staging and the first course of treatment than the short-form database. The TCR database contains information on the date of diagnosis, primary site, stage, treatment, and dates of treatment administration.

Another data source was the NHI database, which contains information on all the diagnostic tests and treatments performed by hospitals in Taiwan^21^. Patient data regarding comorbidities, surgical procedures, drugs, the date and duration of therapy from the files of the registry for beneficiaries, ambulatory care expenditures by visits, inpatient expenditures by admissions, details of ambulatory care orders, and details of inpatient orders for 2011–2018 were extracted from the NHI database. Lastly, Taiwan Death Registry containing date of death was used to confirm the survival status.

### Study participants

The inclusion criteria were as follows: (1) patients newly diagnosed with GIST (see Supplementary Document 1 for the TCR) between January 1, 2012, and December 31, 2014; (2) those who had received at least one TKI therapy (imatinib with or without sunitinib or regorafenib). We excluded (1) patients who only had records of sunitinib or regorafenib, (2) patients who started TKI therapy with sunitinib or regorafenib, and (3) patients with no data on tumor status.

The pathological features and resectability of GISTs are the main factors that influence their management^22^. TKI is the standard treatment for patients receiving adjuvant therapy (after surgical resection) or neoadjuvant therapy, and patients with advanced/metastatic tumors. Because neoadjuvant therapy is not the standard treatment for GISTs in Japan and Taiwan, we only analyzed the data of patients who received adjuvant therapy and patients with advanced/metastatic tumors (hereafter adjuvant setting and advanced/metastatic setting, respectively). Based on the courses of imatinib therapy, first-line treatment, and prescription patterns, the two settings were further divided into three groups: the continued, discontinued, and switched groups (see Supplementary Figs. 1, 2, and 3). The patient selection and classification processes are described in Supplementary Documents 2 and 3. The analysis variables are listed in Supplementary Table 1. The order codes for the medication/procedures are shown in Supplementary Tables 2 and 3.

The patients were also divided into older and non-older groups for further analysis. Patients aged ≥ 75 years were considered older patients. This definition was brought forth by the Japan Gerontological Society and the Japan Geriatrics Society because of the increasing life expectancy in most developed countries^23^. Moreover, this age definition has been used in previous studies investigating GIST outcomes^14,24^.

### Statistical analysis

We calculated the frequency (proportion) for categorical variables and the mean (standard deviation) or median (interquartile range) for continuous variables. To compare the characteristics of the older and non-older patients, the patients were dichotomized using an age cut-off of ≥ 75 years^25^. The t-test, Wilcoxon rank-sum test, and chi-squared test were used for hypothesis testing. To compute the time to stop imatinib for patients receiving TKIs, the index date was defined as the date patients started using imatinib to the date of discontinuation, switching, or December 31, 2018, whichever came first. Similarly, the follow-up time for overall survival analysis was from the date patients of first imatinib prescription to the date of all-cause death or December 31, 2018, whichever came first. Daily imatinib exposure over the entire treatment period was generated using all the imatinib prescriptions during the index period. Kaplan–Meier curves were generated and compared using log-rank tests. To evaluate the association between analysis variables and the time to stop imatinib or the time to decease, the multivariable Cox regressions were adapted to compute adjusted HRs and 95% confidence intervals CIs. All analyses were performed using Stata 14.1 (StataCorp LLC, College Station, TX, USA) and SAS 9.4 (SAS Institute Inc., Cary, NC, USA).

### Ethics declarations

This research was conducted according to the guidelines of the 1964 Declaration of Helsinki and its later amendments. This research was approved by the Research Ethics Committee of National Health Research Institutes (EC109010D4-E) and National Cancer Center Institutional Review Board (2017-379). The requirement for patient consent was waived by both committees because the data were obtained from de-identified databases.

## Results

### Patient characteristics and overall TKI use

**Figure 1:**
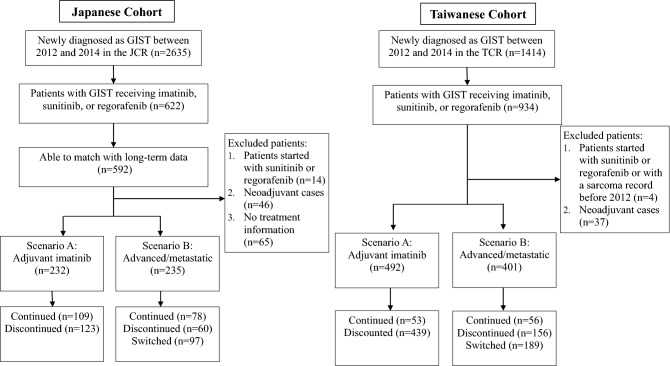
Patient selection flowchart in the JCR and TCR. Details of the patient selection process for each country are explained in the figure. *GIST* Gastrointestinal stromal tumor, *JCR* Japanese Cancer Registry, *TCR* Taiwan Cancer Registry.

Overall, 2635 patients with GISTs from JCR database and 1414 from the TCR database were identified. In the JCR database, 232 patients were in the adjuvant setting, while 235 were in the advanced/metastatic setting. In the TCR database, 492 patients were in the adjuvant setting, while 401 were in the advanced/metastatic setting (Fig. 1).

**Figure 2:**
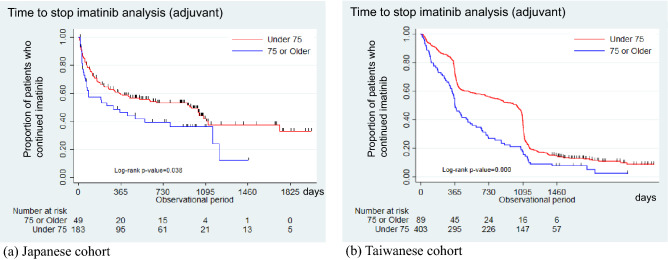
Time to stop imatinib in the older and non-older patients who received adjuvant treatment. (**a**) Japanese cohort. (**b**) Taiwanese cohort.

**Figure 3:**
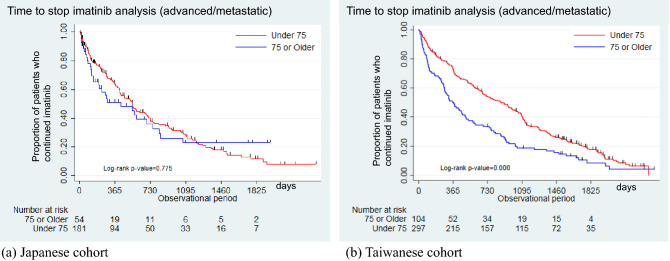
Time to stop imatinib in the older and non-older patients with advanced/metastatic tumors. (**a**) Japanese cohort. (**b**) Taiwanese cohort.

The baseline characteristics of the adjuvant and advanced/metastatic settings are shown in Table 1. The most common primary sites were the stomach (68.5% for the JCR and 45.1% for the TCR in the adjuvant setting; 41.3% and 42.4% in the advanced/metastatic setting, respectively), followed by the small intestine/duodenum (23.3% for the JCR and 45.1% for the TCR in the adjuvant setting, and 35.7% and 34.2% in the advanced/metastatic setting, respectively). The JCR patients were older than the TCR patients (66 vs. 61 years in the adjuvant setting; 66 vs. 63 years in the advanced/metastatic setting, respectively). The median time to stop imatinib for the adjuvant setting was 788 and 783.5 days for the JCR and TCR patients, respectively, and 541 and 678 days for the advanced/metastatic settings, respectively (Figs. 2, 3).

**Table 1:** Baseline characteristics of patients with GISTs in the JCR and TCR databases.

Characteristics	JCR database				TCR database			
	Adjuvant		Advanced/metastatic		Adjuvant		Advanced/metastatic	
	(n = 232)		(n = 235)		(n = 492)		(n = 401)	
Male	108	(46.6)	149	(63.4)	259	(52.6)	249	(62.1)
Age (median [IQR], years)	66	(56, 73)	66	(57, 74)	61	(52, 72)	63	(53, 75)
**Tumor size (cm)**								
≤ 5	74	(31.9)	45	(19.1)	44	(8.9)	36	(9.0)
> 5– ≤ 10	97	(41.8)	79	(33.6)	125	(25.4)	67	(16.7)
> 10	47	(20.3)	70	(29.8)	98	(19.9)	103	(25.7)
Unknown	14	(6.0)	41	(17.4)	225	(45.7)	195	(48.6)
**Mitotic count**								
Low (< 5)	–	–	–	–	112	(22.8)	50	(12.5)
High (> 5)	–	–	–	–	241	(49.0)	135	(33.7)
Unknown	–	–	–	–	139	(28.3)	216	(53.9)
**Stage**								
I	48	(20.7)	20	(8.5)	22	(4.5)	11	(2.7)
II	67	(28.9)	29	(12.3)	77	(15.7)	26	(6.5)
III	103^a^	(44.4)^a^	54	(23.0)	170^a^	(34.5)^a^	61	(15.2)
IV			104	(44.3)			142	(35.4)
Unknown	14	(6.0)	28	(11.9)	223	(45.3)	161	(40.1)
**Distant metastasis**								
No	222	(95.7)	109	(46.4)	281	(57.1)	127	(31.7)
Yes	0	(0)	109	(46.4)	0	(0)	141	(35.2)
Unknown	10	(4.3)	17	(7.2)	211	(42.9)	133	(33.2)
**Year of diagnosis**								
2012	46	(19.8)	50	(21.3)	164	(33.3)	138	(34.4)
2013	105	(45.3)	94	(40.0)	151	(30.7)	123	(30.7)
2014	81	(34.9)	91	(38.7)	177	(36.0)	140	(34.9)
**Primary site**								
Stomach	159	(68.5)	97	(41.3)	222	(45.1)	170	(42.4)
Small intestine/duodenum	54	(23.3)	84	(35.7)	222	(45.1)	137	(34.2)
Others	19	(8.3)	54	(23.0)	48	(9.7)	94	(23.2)
**Treatment group**								
Continued	109	(47.0)	78	(33.3)	53	(10.8)	56	(14.0)
Discontinued	123	(53.0)	60	(25.5)	439	(89.2)	156	(38.9)
Switched	0	(0)	97	(41.3)	0	(0)	189	(47.1)
**Time to stop imatinib, days**								
Median, 95% CI	788	(361, 1040)	541	(416, 616)	783.5	(573, 993)	685	(580, 863)
**Starting dosage (imatinib, mg)**								
≤ 300	20	(8.6)	29	(12.3)	64	(13.0)	58	(14.4)
≥ 400	212	(91.4)	206	(57.7)	428	(87.0)	343	(85.5)
Imatinib dose over the entire treatment period (median [IQR], mg/day)	300	(200, 400)	300	(300, 400)	400	(400, 400)	400	(400, 400)
**Data duration, days**								
Mean, SD	1051.1	(571.6)	1127.5	(710.3)	1830.1	(551.9)	1387.9	(734.0)
Median, IQR	1008	(593, 1443)	1050	(487, 1726)	1901	(1602.5, 2237)	1566	(778, 1974)

*GISTs* Gastrointestinal stromal tumors, *JCR* Japanese Cancer Registry, *TCR* Taiwan Cancer Registry, *CI* Confidence interval, *IQR* Interquartile range, *SD* Standard deviation.

^a^Stages III and IV are combined in the adjuvant setting due to the data regulation policy in Japan.

### Adjuvant settings

In the adjuvant setting, fewer older patients received the standard dose of imatinib (400 mg/day) than the non-older patients in both the Japanese and Taiwanese cohorts (Table 2). The median time to stop imatinib was shorter in the older patients in both cohorts (JCR: 301 vs. 975 days, *p* = 0.038; TCR: 366 vs. 1028 days, *p* < 0.001, Fig. 2). The adjusted hazard ratios (HRs) using the Cox regression analysis were 1.51 (95% CI 0.98–2.33; *p* = 0.065) in the JCR and 1.70 in the TCR (95% CI 1.33–2.16; *p* < 0.0001) (Supplementary Table 4). The adjusted HR after adding the mitotic count and Charlson comorbidity index in the TCR analysis was 1.48 (95% CI 1.14–1.92; *p* = 0.003; Supplementary Table 5). When comparing overall survival status using TCR cohort (Supplementary Fig. 4 and Supplementary Table 6), we found that older patients had significantly worse survival than the non-older patients (*p* < 0.001). The adjusted HR showed that older patients were 3.78 times (95% CI 2.23–6.41, *p* < 0.001) more likely to die than non-older patients, and patients with comorbidities were 3.29 times (95% CI 1.92–5.62, *p* < 0.001) of mortality risk.

**Table 2:** Demographics of patients in the adjuvant setting after stratification by age.

Demographics	JCR database				*p* value	TCR database				*p* value
	< 75		≥ 75			< 75		≥ 75		
	(n = 183)		(n = 49)			(n = 403)		(n = 89)		
Male	82	(44.8)	26	(53.1)	0.304	210	(52.1)	49	(55.1)	0.614
Age (median [IQR], years)	62.0	(54, 69)	78.0	(77, 80)		58	(50, 65)	78	(76, 83)	
**Tumor size (cm)**^**a**^					0.128					0.582
≤ 5	55	(30.1)	19	(38.8)		38	(9.4)	6	(6.7)	
> 5– ≤ 10	83	(45.4)	14	(28.6)		103	(25.6)	22	(24.7)	
> 10	35	(19.1)	12	(24.5)		76	(18.9)	22	(24.7)	
**Mitosis**										0.198
Low	–	–	–	–		86	(21.3)	26	(29.2)	
High	–	–	–	–		204	(50.6)	37	(41.6)	
Unknown	–	–	–	–		113	(28.0)	26	(29.2)	
**Stage**^**a**^					0.440					0.171
I	40	(21.9)	7–9^b^			16	(4.0)	6	(6.7)	
II	52	(28.4)	15	(30.6)		57	(14.1)	20	(22.5)	
III/IV	81	(44.3)	22	(44.9)		145	(35.9)	25	(28.1)	
**Year of diagnosis**					0.932					0.259
2012	36	(19.7)	10	(20.4)		130	(32.3)	34	(38.2)	
2013	82	(44.8)	23	(46.9)		130	(32.3)	21	(23.6)	
2014	65	(35.5)	16	(32.7)		143	(35.5)	34	(38.2)	
**Primary site**					0.831					0.050
Stomach	125	(68.3)	34	(69.4)		172	(42.7)	50	(56.2)	
Others	68	(31.7)	15	(30.6)		231	(57.4)	39	(43.8)	
**Treatment group**					0.106					0.137
Continued	91	(49.7)	18	(36.7)		55	(13.6)	7	(7.9)	
Discontinued	92	(50.3)	31	(63.3)		348	(86.4)	82	(82.1)	
**Time to stop imatinib, days**										
Median, 95% CI	975	(429, 1099)	301	(70,1160)		1028	(793, 1082)	366	(334, 505)	
Starting dosage (imatinib, mg)					< 0.001					0.003
≤ 300	7–9^b^		11	(22.5)		44	(10.9)	20	(22.5)	
400	174–176^b^		38	(77.6)		359	(89.1)	69	(77.5)	
Imatinib dose over the entire treatment period (median [IQR], mg/day)	300	(300, 400)	200	(200, 300)	< 0.001	400	(400, 400)	400	(300, 400)	< 0.001
**Survival status during the follow-up period**										< 0.001
Alive	–	–	–	–		370	(91.8)	54	(60.7)	
Dead	–	–	–	–		33	(8.2)	35	(39.3)	
Median survival time^c^	–	–	–	–		NR		NR		

*JCR* Japanese Cancer Registry, *TCR* Taiwan Cancer Registry, *CI* Confidence interval, *IQR* Interquartile range, *SD* Standard deviation.

^a^Unknown is removed from staging and tumor size.

^b^Any patient group with a size below 10 patients is masked due to the data regulation policy in Japan.

^c^NR: Not reached.

### Advanced/metastatic settings

In the advanced/metastatic settings, most of the older patients started with lower doses of imatinib (< 400 mg/day) than the non-older (JCR: 29.6% vs. 7.2%, *p* < 0.001; TCR: 32.6% vs. 8.1%, *p* < 0.001, Table 3). The median time to stop imatinib was shorter in the older patients than in the non-older (366.5 vs. 884, *p* < 0.001) in the Taiwanese cohort; this difference was not statistically significant in the Japanese patients (423 vs. 542, *p* = 0.775) (Fig. 3). The older patients were more likely to discontinue TKI therapy than the non-older patients when the continued and switched groups were combined into one and compared with the discontinued group (JCR: 35.2% vs. 22.7%, *p* = 0.076; TCR: 62.5% vs. 30.6%, *p* < 0.001). After adjustments for potential confounding variables, the older patients with advanced/metastatic GISTs were more likely to discontinue TKI therapy than the non-older patients (JCR: adjusted HR = 1.17; 95% CI 0.77–1.79; *p* = 0.458; TCR: adjusted HR = 1.58; 95% CI 1.22–2.05; *p* = 0.001; Supplementary Table 4). The adjusted HR after adding the mitotic count and Charlson comorbidity index in the TCR analysis was 1.59 (95% CI 1.23–2.07; *p* = 0.001; Supplementary Table 5). In terms of overall survival status, older patients showed significantly worse prognosis than the non-elderly in the Taiwanese cohort (median survival days: 815 [95% CI 604–1069] vs. 1965 [95% CI 1698–2378] days, *p* < 0.001). The adjusted HR with available covariates showed that older patients were 2.53 times (95% CI 1.85–3.45, *p* < 0.001) more likely to die than non-older patients (Supplementary Fig. 4 and Supplementary Table 6).

**Table 3:** Demographics of patients in the advanced/metastatic setting after stratification by age.

Demographics	JCR database				*p* value	TCR database				*p* value
	< 75		≥ 75			< 75		≥ 75		
	(n = 181)		(n = 54)			(n = 297)		(n = 104)		
Male	116	(64.1)	33	(61.1)	0.690	190	(64.0)	59	(56.7)	0.190
Age (median [IQR], years)	63	(55, 69)	79.0	(77, 82)		58	(50, 65)	81	(77, 85)	
**Tumor size (cm)**^**a**^					0.666					0.300
≤ 5	34	(18.8)	11	(20.4)		24	(8.0)	12	(11.6)	
> 5– ≤ 10	56	(30.9)	23	(42.6)		48	(16.2)	19	(18.3)	
> 10	54	(29.8)	16	(29.6)		83	(27.9)	20	(19.2)	
**Mitosis**										0.120
Low	–	–	–	–		39	(13.1)	11	(10.6)	
High	–	–	–	–		107	(36.0)	28	(26.9)	
Unknown	–	–	–	–		151	(50.8)	65	(62.5)	
**Stage**^**a**^					0.873					0.005
I	14	(7.7)	4–6^b^			4	(1.3)	7	(6.7)	
II	22	(12.2)	7–9^b^			19	(6.4)	7	(6.7)	
III	40	(22.1)	14	(25.9)		40	(13.5)	21	(20.2)	
IV	81	(44.8)	23	(42.6)		116	(39.1)	26	(25.0)	
**Distant metastasis**					0.334					0.020
Yes	87	(48.1)	22	(40.7)		116	(39.1)	25	(24.0)	
**Year of diagnosis**					0.637					0.148
2012	37	(20.4)	13	(24.1)		98	(33.0)	40	(38.5)	
2013	71	(39.2)	23	(42.6)		99	(33.3)	24	(23.1)	
2014	73	(40.3)	18	(33.3)		100	(33.7)	40	(38.5)	
**Primary site**					0.100					0.166
Stomach	69	(38.1)	28	(51.9)		123	(41.4)	47	(45.2)	
Small intestine/duodenum	71	(39.2)	13	(24.1)		109	(36.7)	28	(26.9)	
Others	41	(22.7)	13	(24.0)		65	(21.9)	29	(27.9)	
**Treatment group**					0.012					< 0.001
Continued	56	(30.9)	22	(40.7)		46	(15.5)	10	(9.6)	
Discontinued	41	(22.7)	19	(35.2)		91	(30.6)	65	(62.5)	
Switched	84	(46.4)	13	(24.1)		160	(53.9)	29	(27.9)	
**Treatment group**					0.076					< 0.001
Continued + switched	140	(77.3)	35	(64.8)		206	(69.4)	39	(37.5)	
Discontinued	41	(22.7)	19	(35.2)		91	(30.6)	65	(62.5)	
**Time to stop imatinib, days**										
Median, 95% CI	542	(416, 694)	423	(196, 752)		884	(683, 1021)	366.5	(272, 491)	
**Starting dosage (imatinib, mg)**					< 0.001					< 0.001
≤ 300	13	(7.2)	16	(29.6)		24	(8.1)	34	(32.6)	
≥ 400	168	(92.8)	38	(70.4)		273	(91.9)	70	(67.3)	
Imatinib dose over the entire treatment period (median [IQR], mg/day)	400	(300, 400)	300	(200, 300)	< 0.001	400	(400, 400)	400	(300, 400)	< 0.001
**Survival status during the follow-up period**										< 0.001
Alive	–	–	–	–		158	53.2	23	22.1	
Dead	–	–	–	–		139	46.8	81	77.9	
Median survival time, days						1965		815		

*JCR* Japanese Cancer Registry, *TCR* Taiwan Cancer Registry, *CI* Confidence interval, *IQR* Interquartile range, *SD* Standard deviation.

^a^Unknown is removed from staging and tumor size.

^b^Any patient group with a size below 10 patients is masked due to the data regulation policy in Japan.

## Discussion

Using nationwide datasets, we assessed the long-term use of TKIs for patients with GISTs. Our results showed that imatinib was discontinued earlier in both the adjuvant or advanced/metastatic settings, compared to the guidelines’ recommendation or clinical trials, respectively. Moreover, the starting doses of imatinib were lower than those recommended by the Asian consensus guidelines in some patients^10^. These trends were prominent in patients aged ≥ 75 years. To the best of our knowledge, this is the first study to describe the long-term follow-up of TKI therapy in older patients with GISTs in Japan and Taiwan.

GIST is the most common rare cancer among the older population, but it is unknown whether established treatment in younger patients with GISTs is applicable to older patients. We used 75 years as the cut-off age for older patients in this study based on the recommendations of the Japan Gerontological Society and the Japan Geriatrics Society, which may reflect the aging situations of both countries^23,26^. Our study is the first retrospective cohort study using both national registry and health insurance claims data, which were used to identify treatment patterns for older patients with GISTs in the real world.

The GIST clinical practice guidelines recommend 3-year adjuvant therapy for high-risk GISTs^8,10,22,27^. Our results showed that the older patients with GISTs tended to receive a lower initial imatinib dose and for a shorter duration than the non-older patients. Previous studies have reported a higher imatinib discontinuation rate in older patients than in non-older patients due to patient choice, drug toxicity, or deterioration of physical function, leading to more postoperative complications^13,28^. Although 3-year imatinib therapy and a standard dose of 400 mg/day are recommended in adjuvant or advanced/metastatic settings, a shorter duration of imatinib therapy and a low starting dose may ensure dose optimization in older patients with confronting factors, such as comorbidities.

Older patients had worse survival as compared to non-older in both settings (Supplementary Fig. 4), which were consistent with the previous reports^13,14^. The results from Farag et al.^14^ showed a median OS was 34 months in elderly patients and 59 months in non-elderly patients for metastatic settings, whereas our results from Taiwanese patients were 27.2 months (815 days; 95% CI 20.1–35.6 months) and 65.5 months (1965 days; 95% CI 56.6–79.3 months), correspondingly. Our older patients have a shorter median survival time. In our OS multivariable analysis of advanced/metastatic setting (Supplementary Table 6), age and gender were two significant factors associated with OS. While the patients’ gender distributions were similar between the two studies, future study may be conducted to investigate factors associated with medication management.

Analysis of the time to stop imatinib in patients who received adjuvant therapy revealed a unique shape in each country (Fig. 2): a flat line after 3 years in the Japanese cohort and stepwise drop at 1 year and 3 years in the Taiwanese cohort. In the Japanese cohort, a significant drop was observed at around 3 years due to the guidelines’ recommendations. The flat lines after 3 years may have been affected by the small sample size. The two drops in the Taiwanese cohort around 1 year and 3 years may be due to the change in the reimbursement policy during the study period: adjuvant imatinib was reimbursed for only 1 year before September 2013 and for 3 years after September 2013. In addition, the Japanese cohort had more censored cases within 4 years of follow-up compared to the Taiwanese cohort. This may be related to a limitation of the Japanese dataset, whereby the data is unable to capture patients who transferred to other hospitals.

Although we used the same data collection and analysis methods for the data from two cancer registries and two insurance reimbursement systems, some results showed different trends. The GIST clinical practice guidelines recommend TKI therapy, including imatinib and sunitinib, for advanced or metastatic GISTs until drug resistance occurs^10,22,27,29,30^. The older patients in the Taiwanese cohort received imatinib for a shorter duration than the non-older patients; this finding was different in the Japanese cohort. TKI discontinuation in the first line showed similar trends between Japan and Taiwan with a marginally statistical significance. While Yang et al. reported that older patients with GISTs may have different treatment patterns from non-older patients due to comorbidities or lower physical functions, Farag et al. reported that older patients with GISTs received the same treatment as non-older patients with GISTs^13,14^. Both countries did not have similar trends; therefore, the difference between the two countries needs to be explored meticulously in the near future. Our results showed that older patients started imatinib at lower doses than the non-older in both countries. A similar proportion of dose reduction was observed previously in older patients^11,14^. Numerous efforts have been made to optimize doses in older patients with GISTs.

Our study had several limitations. We were unable to follow-up the Japanese patients who moved to other hospitals because we collected the health claims data from each facility without identification. This may be associated with censored cases of the Japanese cohort within 3 years of imatinib treatment^22^. Also, the results of the Japanese cohort were unable to analyze some outcomes, such as survival status or disease progression, or adjust for some covariates, such as mitotic count or comorbidities, because these variables were unavailable in the data. In the TCR database, mitotic count, stage, metastasis status, and tumor size were only available for the long-form registry (gastric and colorectal GISTs) and not the short-form registry (e.g., small intestinal/duodenal GISTs). Finally, our study focused on high-risk GIST patients who received imatinib because we aimed to describe the patterns of imatinib use. Although imatinib is a standard treatment option for high-risk GIST patients, it can be modified based on the physician’s judgment of the patient’s condition. The indications of imatinib for individual patients remain unclear in the database. Although there is a possibility that patients may receive TKIs, which are not reimbursed, we assume that the numbers may be insignificant and could be ignored. Therefore, because of the high cost of cancer medications, most patients would pursue treatment through cancer care system in both countries. Regarding PFS, information of treatment prognosis was unavailable in our analysis database. However, imatinib is fully reimbursed for unresectable or metastatic GISTs, and for the adjuvant setting, it is reimbursed for 1–3 years in Taiwan. In Japan, imatinib is reimbursed for both settings of patients. Therefore, discontinuation of imatinib can be considered as a surrogate observation for unfavorable outcomes, including disease progression, in real-world data studies. Furthermore, assuming non-differential outcome misclassification implies that the proportions of imatinib discontinuation due to adverse events are similar between older and non-older groups, our analysis of time-to-discontinuation may be a feasible surrogate of PFS in both settings of Japanese patients and advanced/metastatic setting of Taiwan patients.

In summary, our study investigated the long-term use of imatinib in patients with GISTs in Japan and Taiwan according to age. Older patients with GISTs receive a lower initial dose and a shorter duration of imatinib treatment than non-older patients. Future research should explore detailed reasons for the discontinuation of treatment in older patients. Healthcare providers should be encouraged to offer standard treatment for maximizing patient outcomes.

## Supplementary Information

Supplementary Information.

## Data Availability

The Japanese datasets analyzed during the study are available from the authors on reasonable request. The Taiwanese data that support the findings of this study are available from Health and Welfare Data Science Center, Ministry of Health and Welfare but restrictions apply to the availability of these data, which were used under license for the current study, and so are not publicly available. Data regarding the Taiwanese cohort are however available from the authors upon reasonable request and with permission of Health and Welfare Data Science Center, Ministry of Health and Welfare, Taiwan.

## References

[1] Japanese Society of Medical Oncology (2018). Clinical Oncology Update-Essentials for the Medical Oncologists.

[2] Chiang NJ, Chen LT, Tsai CR, Chang JS (2014). The epidemiology of gastrointestinal stromal tumors in Taiwan, 1998–2008: A nation-wide cancer registry-based study. BMC Cancer.

[3] Søreide K (2016). Global epidemiology of gastrointestinal stromal tumours (GIST): A systematic review of population-based cohort studies. Cancer Epidemiol..

[4] Kelly CM, Gutierrez Sainz L, Chi P (2021). The management of metastatic GIST: Current standard and investigational therapeutics. J. Hematol. Oncol..

[5] Demetri GD (2002). Efficacy and safety of imatinib mesylate in advanced gastrointestinal stromal tumors. N. Engl. J. Med..

[6] Casali PG (2021). Final analysis of the randomized trial on imatinib as an adjuvant in localized gastrointestinal stromal tumors (GIST) from the EORTC Soft Tissue and Bone Sarcoma Group (STBSG), the Australasian Gastro-Intestinal Trials Group (AGITG), UNICANCER, French Sarcoma Group (FSG), Italian Sarcoma Group (ISG), and Spanish Group for Research on Sarcomas (GEIS). Ann. Oncol..

[7] Eisenberg BL (2013). The SSG XVIII/AIO trial: Results change the current adjuvant treatment recommendations for gastrointestinal stromal tumors. Am. J. Clin. Oncol..

[8] Joensuu H (2012). One vs three years of adjuvant imatinib for operable gastrointestinal stromal tumor a randomized trial. JAMA.

[9] DeMatteo RP (2009). Adjuvant imatinib mesylate after resection of localised, primary gastrointestinal stromal tumour: A randomised, double-blind, placebo-controlled trial. Lancet.

[10] Koo DH (2016). Asian consensus guidelines for the diagnosis and management of gastrointestinal stromal tumor. Cancer. Res. Treat..

[11] Italiano A (2013). Treatment of advanced gastrointestinal stromal tumors in patients over 75 years old: Clinical and pharmacological implications. Target Oncol..

[12] Kim JH (2019). Long-term survival outcome with tyrosine kinase inhibitors and surgical intervention in patients with metastatic or recurrent gastrointestinal stromal tumors: A 14-year, single-center experience. Cancer Med..

[13] Yang Z (2019). Clinicopathological outcomes and prognosis of elderly patients (≥ 65 years) with gastric gastrointestinal stromal tumors (GISTs) undergoing curative-intent resection: A multicenter data review. J. Gastrointest. Surg..

[14] Farag S (2017). Elderly patients with gastrointestinal stromal tumour (GIST) receive less treatment irrespective of performance score or comorbidity—A retrospective multicentre study in a large cohort of GIST patients. Eur. J. Cancer.

[15] Corless CL (2014). Pathologic and molecular features correlate with long-term outcome after adjuvant therapy of resected primary GI stromal tumor: The ACOSOG Z9001 trial. J. Clin. Oncol..

[16] von Elm E (2014). The strengthening the reporting of observational studies in epidemiology (STROBE) statement: Guidelines for reporting observational studies. Int. J. Surg..

[17] Okuyama A, Tsukada Y, Higashi T (2021). Coverage of the hospital-based cancer registries and the designated cancer care hospitals in Japan. Jpn. J. Clin. Oncol..

[18] Iwamoto M, Nakamura F, Higashi T (2016). Monitoring and evaluating the quality of cancer care in Japan using administrative claims data. Cancer Sci..

[19] Watanabe T (2018). Quality indicators for cervical cancer care in Japan. J. Gynecol. Oncol..

[20] Chiang CJ (2015). Quality assessment and improvement of nationwide cancer registration system in Taiwan: A review. Jpn. J. Clin. Oncol..

[21] Hsieh CY (2019). Taiwan’s National Health Insurance Research Database: Past and future. Clin. Epidemiol..

[22] Casali PG (2018). Gastrointestinal stromal tumours: ESMO-EURACAN Clinical Practice Guidelines for diagnosis, treatment and follow-up. Ann. Oncol..

[23] Ouchi Y (2017). Redefining the elderly as aged 75 years and older: Proposal from the Joint Committee of Japan Gerontological Society and the Japan Geriatrics Society. Geriatr. Gerontol. Int..

[24] Yamano T (2017). Influence of age and comorbidity on prognosis and application of adjuvant chemotherapy in elderly Japanese patients with colorectal cancer: A retrospective multicentre study. Eur. J. Cancer..

[25] Tokuda Y, Hinohara S (2008). Geriatric nation and redefining the elderly in Japan. Int. J. Gerontol..

[26] Lin YY, Huang CS (2016). Aging in Taiwan: Building a society for active aging and aging in place. Gerontologist.

[27] Yeh C-N (2012). Clinical practice guidelines for patients with gastrointestinal stromal tumor in Taiwan. World J. Surg. Oncol..

[28] Raut CP (2018). Efficacy and tolerability of 5-year adjuvant imatinib treatment for patients with resected intermediate- or high-risk primary gastrointestinal stromal tumor: The PERSIST-5 clinical trial. JAMA Oncol..

[29] Nishida T, Blay JY, Hirota S, Kitagawa Y, Kang YK (2016). The standard diagnosis, treatment, and follow-up of gastrointestinal stromal tumors based on guidelines. Gastric Cancer.

[30] Nishida T (2008). Clinical practice guidelines for gastrointestinal stromal tumor (GIST) in Japan: English version. Int. J. Clin. Oncol..

