# Supplementary material for: Imatinib use for gastrointestinal stromal tumors among older patients in Japan and Taiwan
Source: Sci Rep. 2022 Dec 28;12:22492. doi: 10.1038/s41598-022-27092-z (PMC9797546; doi:10.1038/s41598-022-27092-z)
Supplement: Supplementary file 1 — Supplementary Information. [file 41598_2022_27092_MOESM1_ESM.docx]

Article

Journal name: *Scientific Reports*

Title: Imatinib Use for Gastrointestinal Stromal Tumors among Older Patients in Japan and Taiwan

Yuichi Ichinose^1*^, Yi-Hsin Yang^2*^, Hui-Jen Tsai^2^, Ru-Yu Huang^2^, Takahiro Higashi^1,3^, Toshirou Nishida^4,5^, Li-Tzong Chen^2,6,7^

^1^ Division of Health Services Research, Institute for Cancer Control, National Cancer Center, 5-1-1, Tsukiji, Chuo-ku, Tokyo 104-0045, Japan

^2^ National Institute of Cancer Research, National Health Research Institutes, No.367, Sheng-Li Rd., North District, Tainan 70456, Taiwan

^3^ Department of Public Health and Health Policy, School of Medicine, the University of Tokyo, 7-3-1, Hongo, Bunkyo-ku, Tokyo 113-0033, Japan.

^4^ National Cancer Center Hospital, 5-1-1, Tsukiji, Chuo-ku, Tokyo 104-0045, Japan

^5^ Department of Surgery, Japan Community Health Care Organization Osaka Hospital, 4-2-78, Fukushima, Fukushima-ku, Osaka 553-0003, Japan

^6^ Department of Internal Medicine, Kaohsiung Medical University Hospital, Kaohsiung Medical University, Kaohsiung, No.100, Tzyou 1st Rd., Sanmin Dist., Kaohsiung City 80756, Taiwan

^7^ Department of Oncology, National Cheng Kung University Hospital, National Cheng Kung University, No.138, Sheng Li Rd., North Dis., Tainan70403, Taiwan

Corresponding Authors:

Yuichi Ichinose

Division of Health Services Research, Institute for Cancer Control,

National Cancer Center

5-1-1, Tsukiji, Chuo-ku, Tokyo 104-0045, Japan

Email: [yuichino@ncc.go.jp](mailto:yuichino@ncc.go.jp)

Telephone: 81-3-3542-2511

Yi-Hsin Yang

National Institute of Cancer Research, National Health Research Institutes

No.367, Sheng-Li Rd., North District, Tainan 70456, Taiwan

Email: [yhyang@nhri.edu.tw](mailto:yhyang@nhri.edu.tw)

Telephone: 886-6-700-0123

Supplementary Materials

[Supplementary Table 1. Findings of the data analyses and variables 5](#_Toc120535942)

[Supplementary Table 2. List of the gastrointestinal stromal tumor-related surgeries 13](#_Toc120535943)

[Supplementary Table 3. List of tyrosine kinase inhibitors 17](#_Toc120535944)

[Supplementary Table 4. Multivariable Cox regression analysis of the time to stop imatinib 18](#_Toc120535945)

[Supplementary Table 5. Multivariable Cox regression analysis of the time to stop imatinib with additional variables (Taiwan only) 19](#_Toc120535946)

[Supplementary Table 6. Multivariable Cox regression analysis for overall survival (Taiwan only) 22](#_Toc120535947)

[Supplementary Document 1. GIST registry in the Taiwan Cancer Registry 24](#_Toc120535948)

[Supplementary Document 2. Definitions of the setting and prescription patterns 26](#_Toc120535949)

[Supplementary Document 3. Detailed steps of the extraction of the Japanese and Taiwanese data 28](#_Toc120535950)

[Supplementary Document 4. Reimbursement for the GIST-targeted therapies in the Taiwan National Health Insurance Program 31](#_Toc120535951)

[Supplementary Figure 1. Diagram of the patient conditions 34](#_Toc120535952)

[Supplementary Figure 2. Illustration of the imatinib treatment pattern groups in the adjuvant setting 35](#_Toc120535953)

[Supplementary Figure 3. Illustration of the imatinib treatment pattern groups in the advanced/metastatic setting 36](#_Toc120535954)

[Supplementary Figure 4. Kaplan Meier analysis for overall survival (Taiwan only) 37](#_Toc120535955)

# Supplementary Table 1. Findings of the data analyses and variables

**Japanese data**

| **Analysis variable** | **Data file name** | **Variable name** | **Description** |
| --- | --- | --- | --- |
| Hospital-Based Cancer Registry (HBCR) Data | | | |
| Sex |  | v50 | 1: Male, 2: Female, 9: Unknown |
| Date of birth |  | v60 | Date of birth |
| Date of diagnosis |  | diag | Date of diagnosis |
| Age at diagnosis |  | v60, diag | We calculated the age at diagnosis by subtracting the date of birth (variable: v60) from the date of diagnosis (variable: diag) in the HBCR data, and then divided those days by 365.25. |
| Class of case (CoC) |  | v180 | We selected the records with a CoC = 2, 3, or 4.  1: diagnosed at the reporting facility but started the first treatment elsewhere  2: diagnosed and started the first treatment at the reporting facility  3: diagnosed at a different facility but started the first cancer treatment at the reporting facility  4: diagnosed and started the first treatment at a different facility and came to the reporting facility later  8: others  9: unknown |
|  |  |  |  |
| Primary site |  | v200 | Based on the ICD-O-3 site code |
| Histology |  | v330 | Based on the ICD-O-3 morphology code  We obtained the records with the morphology codes of 8936/0 and 8936/3. |
| Stage |  | v230, v270 | Based on the Tumor–Node–Metastasis (TNM) Classification of Malignant Tumours, 7th Edition  If the pathologic stage (variable: v270) was available in the HBCR data, this variable was used to obtain the staging information. If the variable was not available, we used the clinical stage (variable: v230) in the HBCR data. |
| Metastasis |  | v310, v320 | Coding as “4”  If the postsurgical metastasis status (variable: v320) was available in the HBCR data, this variable was used to evaluate the metastasis status. If the variable was not available, we used the metastasis status prior to the initiation of treatment (variable: v310) in the HBCR data. |
| Tumor size |  | v240, v280 | Based on the T classification in the TNM Classification of Malignant Tumours, 7^th^ Edition  If the tumor size of the tumor known following the completion of surgical therapy (variable: v280) was available in the HBCR data, this variable was used to obtain the tumor size in the HBCR data. If the variable was not available, we used the tumor size of the tumor known prior to the start of any treatment (variable: v240) in the HBCR data. |
| Mitosis | - | - | There was no available data for the mitotic count in the Japanese data. |
| Diagnostic Procedure Combination Data | | | |
| Procedures (medication/ surgery) | EF file | ef9 | Code to specify medications and procedures.  Lists of index drugs including imatinib, sunitinib, and regorafenib and GIST-related procedures were in Supplementary Table 5 |
| Date of the procedure | EF file | ef24 | Date of the procedure and drug prescription |
| Comorbidities | - | - | NO INFORMATION |
| Days of prescription | EF file | ef21 | Number of days of administration per prescription |
| Dosage | EF file | ef12 | Dosage |

**Taiwanese data**

| **Analysis variable** | **Data file name** | **Variable name** | | **Description** | |
| --- | --- | --- | --- | --- | --- |
| Taiwan Cancer Registry (TCR) | | | | | |
| Diagnosed age | Annual Report File | DIAGAGE | | Age at diagnosis in years | |
| Primary site | Long Form, Short Form | CASITE | | Based on the ICD-O-3 | |
| Class | Long Form, Short Form | Class | | Given that the patients had multiple records, we selected the records with class = 1 or 2.  Class 1: Diagnosed at the reporting hospital and met any of the following criteria:   - Received full or partial first treatment at the reporting hospital - The treatment plan involved no treatment or close observation at the reporting hospital - The patient could not receive treatment or palliative care because they were older or had a severe illness or other medical conditions.   Class 2: Diagnosed at other hospitals and met any of the following criteria:   - Received full or partial first treatment (including palliative care) at the reporting hospital - The first treatment plan was no treatment or close observation at the reporting hospital. | |
| Diagnosed date | Annual Report File | DIAG_DT | | First date of diagnosis | |
| Sex | Annual Report File | ID_S | | 1: Male, 2: Female | |
| Histology | Long Form, Short Form | HIST, HISTBEH | | 89363 | |
| Stage | Long Form, Short Form | CSTAGE, PSTAGE | | I, II, III, IV | |
| Metastasis | Long Form | CM, PM | | Coded as “1” | |
| Surgery | Long Form | OPTYPE | | Codes “10” to “80” were considered as underwent surgery.  Records of the surgical procedure involving the primary site at the reporting hospital:  00: No surgery involving the primary site  10–19: Local tumor destruction  20–29: Local tumor excision  30–80: Tumor excision  90: Unclear surgical approach | |
|  | Short Form | OPTYPE | | Code “90” was considered as underwent surgery.  The surgical procedure involving the primary site at the reporting hospital includes:  00: No surgery involving the primary site  90: Surgery of the primary tumor | |
| Surgery date | Long Form | OPDEF_DT | | The TCR surgery date was used only when no relevant surgery records were found in the NHIRD because the cost was covered by the patient. | |
|  | Short Form | OP_DT | | The earliest surgery date for cancer at the reporting hospital | |
| Tumor size | Long Form | TSIZE_C | | Maximum tumor diameter | |
| Mitosis | Long Form, Short Form | GRADE | | Mitotic rate ≤5/50 high power field (HPF), Low grade; Mitotic rate >5/50 HPF, High grade | |
| National Health Insurance Research Database (NHIRD) | | | | | |
| Date of diagnosis, surgery, or drug prescription | Ambulatory Care Expenditures by Visits | FUNC_DATE | | Date identified as the date of diagnosis, surgery, or drug prescription | |
|  | Inpatient Expenditures by Admissions | IN_DATE | |  |  |
|  | Expenditures for Prescriptions Dispensed at  Contracted Pharmacies | DRUG_DATE | |  |  |
| Comorbidities | Ambulatory Care Expenditures by Visits | ICD9CM_1, ICD9CM_2 | | The Charlson comorbidity index^§^ was identified based on the ICD-9 and ICD-10 diagnosis codes (ICD-10 from 2016). | |
|  | Inpatient Expenditures by Admissions | ICD9CM_1–ICD9CM_5 | |  |  |
| Medication/ surgery | Details of Ambulatory Care Orders | DRUG_NO | | National health insurance order codes;  the index drug included imatinib, sunitinib, and regorafenib.  The GIST-related procedures are listed in Appendix 4. | |
|  | Details of Inpatient Order | ORDER_CODE | |  |  |
|  | Details of Prescriptions Dispensed at Contracted Pharmacies | DRUG_NO | |  |  |
| Drug day | Details of Ambulatory Care Orders | DRUG_DAY | | To obtain the total of the prescription days | |
|  | Inpatient Expenditures by Admissions | E_BED_DAY, S_BED_DAY | |  |  |
|  | Expenditures for Prescriptions Dispensed at  Contracted Pharmacies | DRUG_DAY | |  |  |
| Dose | Details of Ambulatory Care Orders | DRUG_USE, DRUG_FRE, TOTAL_Q, DRUG_DAY | | To calculate the daily dose | |
|  | Details of Inpatient Orders |  |  |  |  |
|  | Details of Prescriptions Dispensed at  Contracted Pharmacies |  |  |  |  |
| Death Registry | | | | |  |
| Date of death | Multiple Cause of Death Data | | D_DATE |  |  |

§ Deyo, R.A., Cherkin, D.C., Ciol, M.A. Adapting a clinical comorbidity index for use with ICD-9-CM administrative databases. *J. Clin. Epidemiol.* **45,**613–619 (1992).

# Supplementary Table 2. List of the gastrointestinal stromal tumor-related surgeries

**Japanese data**

| **Procedure code** | **Procedure** |
| --- | --- |
| 150133810 | Esophagectomy with reconstruction |
| 150134210 | Simple esophagectomy (thoracic esophagus) |
| 150135110 | Esophagectomy with reconstruction (cervical, thoracic, and abdominal procedures) |
| 150135210 | Esophagectomy with reconstruction (thoracic and abdominal procedures) |
| 150161510 | Excision of omental, mesenteric, and retroperitoneal malignant tumor (with intestinal resection) |
| 150161610 | Excision of omental, mesenteric, and retroperitoneal malignant tumor (without intestinal resection) |
| 150165210 | Simple gastrectomy |
| 150166110 | Total gastrectomy (simple) |
| 150168010 | Gastrectomy (malignancy) |
| 150168110 | Total gastrectomy (malignancy) |
| 150181210 | Resection of the small intestine (non-malignant) |
| 150181310 | Resection of small intestinal tumor/diverticulum |
| 150181710 | Colectomy (small region) |
| 150181810 | Hemicolectomy |
| 150181910 | Total/subtotal colectomy (malignant) |
| 150187010 | Excision of rectal tumor (trans-abdominal/transanal) |
| 150187110 | Resection of the rectum |
| 150187210 | Resection of the rectum |
| 150245410 | Low anterior resection of the rectum |
| 150253610 | Excision of esophageal tumor (open thoracic/abdominal) |
| 150271950 | Laparoscopic resection of the small intestine (non-malignant) |
| 150274710 | Laparoscopic resection of esophageal tumor |
| 150277810 | Laparoscopic colectomy (small regional resection or hemicolectomy) |
| 150297310 | Resection of the small intestine (malignant) |
| 150297510 | Ultra-low anterior resection of the rectum (trans-anal coloanal pouch reconstruction) |
| 150317710 | Thoracoscopic esophageal tumor resection |
| 150323210 | Stomach wedge resection |
| 150323310 | Laparoscopic gastrectomy |
| 150323410 | Laparoscopic gastrectomy |
| 150323510 | Laparoscopic gastrectomy |
| 150323610 | Laparoscopic gastrectomy |
| 150323710 | Laparoscopic gastrectomy |
| 150324910 | Laparoscopic colonic tumor resection |
| 150325210 | Laparoscopic rectal resection |
| 150337210 | Proximal simple hemigastrectomy |
| 150337310 | Proximal hemigastrectomy (malignant) |
| 150337810 | Laparoscopic low anterior resection of the rectum |
| 150337910 | Laparoscopic low anterior resection of the rectum |
| 150363710 | Laparoscopic resection of the small intestine (malignant) |
| 150377610 | Laparoscopic stomach wedge resection (with endoscopic assistance) |
| 150377710 | Laparoscopic stomach wedge resection (miscellaneous) |

**Taiwanese data**

| **NHI code** | | **Procedure** |
| --- | --- | --- |
| 75602C | Excision of benign abdominal wall tumor |  |
| 75603B | Excision of malignant abdominal wall tumor |  |
| 75806B | Excision of benign intraabdominal tumor |  |
| 75810B | Excision of malignant intraabdominal tumor |  |
| 73008B | Excision of benign bowel lesion |  |
| 71209B | Esophagectomy |  |
| 71210B | Esophagectomy and reconstruction |  |
| 71211B | Esophagetomy (transcervical or transthoracic) |  |
| 71212B | Excision of esophageal cyst and tumor |  |
| 71215B | Simple excision of esophageal cancer, with lymphadenectomy |  |
| 71222B | Complicated excision of esophageal cancer, with lymphadenectomy |  |
| 71223B | Thoracoscopic excision of esophageal cyst and tumor |  |
| 71224B | Thoracoscopic esophagectomy |  |
| 71219B | Esophagogastric stent for esophagus or cardia cancer |  |
| 72030B | Proximal gastrectomy and esophagectomy and reconstruction |  |
| 72031B | Gastrectomy, total, with splenectomy or partial pancreatectomy |  |
| 72006B | Local excision, ulcer or tumor |  |
| 72007B | Total gastrectomy and reconstruction |  |
| 72009B | Subtotal gastrectomy or hemigastrectomy with gastro-duodenostomy without vagotomy |  |
| 72010B | Subtotal gastrectomy or hemigastrectomy, with vagotomy |  |
| 72032B | Total gastrectomy with LN dissection and reconstruction (any type) |  |
| 72034B | Resection of the retained antrum, post-gastrectomy |  |
| 72043B | Subtotal gastrectomy or hemigastrectomy with gastrojejunostomy without vagotomy |  |
| 72044B | Subtotal gastrectomy or hemigastrectomy with Roux-en-Y gastrojejunostomy without vagotomy |  |
| 72046B | Near total gastrectomy with LN dissection and reconstruction |  |
| 72047B | Radical subtotal gastrectomy with reconstruction |  |
| 72048B | Laparoscopic subtotal gastrectomy |  |
| 73011B | Partial colectomy with anastomosis |  |
| 73012B | Colectomy or radical hemicolectomy with anastomosis of the ascending colon |  |
| 73013B | Left hemicolectomy or sigmoid colectomy |  |
| 73014B | Left hemicolectomy or sigmoid colectomy with anastomosis of the lymph node |  |
| 73015B | Colectomy, total or subtotal-benign |  |
| 73017B | Colectomy, total with proctectomy, with ileostomy |  |
| 73045B | Laparoscopic right colectomy and anastomosis |  |
| 73046B | Laparoscopic anterior resection and anastomosis (sigmoid colon resection)-benign |  |
| 73047B | Colectomy, total or subtotal-malignant |  |
| 73048B | Laparoscopic anterior resection and anastomosis (sigmoid colon resection)-malignant |  |
| 74002B | Appendectomy |  |
| 74004B | Laparoscopic appendectomy |  |
| 74205B | Radical proctectomy with pelvic lymph node dissection |  |
| 74206B | Hartmann’s operation-benign |  |
| 74211B | Extensive excision of sacrococcygeal rectal villous adenoma or malignancy |  |
| 74213B | Restorative proctectomy with coloanal anastomosis |  |
| 74216B | Combined abdominoperineal resection for rectal cancer |  |
| 74217B | Proctosigmoidectomy with pull-through coloanal anastomosis |  |
| 74220B | Posterior proctotomy, transsacrococcygeal resection of malignant tumor (including Kraske and Mason’s procedure) |  |
| 74223B | Hartmann’s operation – malignant |  |
| 75002B | Partial hepatectomy |  |
| 75003B | Segmental hepatectomy-one segment |  |
| 75004B | Segmental hepatectomy-two segments |  |
| 75005B | Segmental hepatectomy-three segments |  |
| 75015B | Right lobectomy |  |
| 75016B | Left lobectomy |  |
| 75017B | Extended right lobectomy |  |
| 75018B | Extended left lobectomy |  |
| 75807B | Excision of retroperitoneal tumor, benign |  |
| 75811B | Excision of retroperitoneal tumor, malignant with retroperitoneal lymphadenectomy |  |
| 74210B | Excision, sacrococcygeal tumor, benign |  |
| 72024B | Excision of duodenum tumor |  |
| 73010B | Resection of the small bowel, with anastomosis |  |
| 73010B | Resection of the small bowel, with anastomosis |  |
| 73030B | Anastomosis of the bowel entero-enterostomy or duodeno-enterostomy |  |
| 73031B | Anastomosis of the bowel-ileocolostomy, side-to-side anastomosis |  |
| 73032B | Anastomosis of the bowel-for intestinal atresia or stenosis |  |
| 75412B | Pancreaticoduodenectomy, Whipple type with reconstruction (including partial gastrectomy) |  |
| 75417B | Pancreaticoduodenectomy, Whipple type with reconstruction (pylorus-sparing Whipple operation) |  |

# Supplementary Table 3. List of tyrosine kinase inhibitors

**Japanese data**

| **Code** | **Drug name** |
| --- | --- |
| 610451029 | Imatinib |
| 620002511 |  |
| 622287101 |  |
| 622291501 |  |
| 622292801 |  |
| 622298801 |  |
| 622306801 |  |
| 622340201 |  |
| 622340301 |  |
| 622348701 |  |
| 622357601 |  |
| 622357701 |  |
| 622375401 |  |
| 622380201 |  |
| 622388501 |  |
| 622389601 |  |
| 622411601 |  |
| 622411701 |  |
| 622414301 |  |
| 622417501 |  |
| 622436501 |  |
| 622436601 |  |
| 622437501 |  |
| 622457401 |  |
| 622496001 |  |
| 622225801 | Regorafenib |
| 620006801 | Sunitinib |

**Taiwanese data**

| **National Health Insurance** **code** | **Drug name** |
| --- | --- |
| AC57915100 | Imatinib |
| AC58288100 |  |
| AC59272100 |  |
| AC59680100 |  |
| B023291100 |  |
| B024027100 |  |
| BB26613100 |  |
| BB26614100 |  |
| BC23291100 |  |
| BC24027100 |  |
| BC26383100 |  |
| BC27372100 |  |
| BC27373100 |  |
| BC26168100 | Regorafenib |
| B024593100 | Sunitinib |
| B024594100 |  |
| B024595100 |  |
| BC24593100 |  |
| BC24594100 |  |
| BC24595100 |  |

# Supplementary Table 4. Multivariable Cox regression analysis of the time to stop imatinib

| **Adjuvant setting** | | | | | | | | | | |
| --- | --- | --- | --- | --- | --- | --- | --- | --- | --- | --- |
|  | Japan | | | | | Taiwan | | | | |
|  | Unadjusted  HR | Adjusted HR | 95% CI | | P-value | Unadjusted  HR | Adjusted HR | 95% CI | | P-value |
|  |  |  | LL | UL |  |  |  | LL | UL |  |
| Age: ≥75 vs. <75 years | 1.53 | 1.51 | 0.98 | 2.33 | 0.065 | 1.69 | 1.70 | 1.33 | 2.16 | <.0001 |
| Sex: female vs. male | - | 1.12 | 0.78 | 1.60 | 0.550 | - | 0.97 | 0.80 | 1.17 | 0.719 |
| Year of diagnosis: 2013 vs. 2012 | - | 0.96 | 0.60 | 1.52 | 0.860 | - | 0.87 | 0.69 | 1.09 | 0.227 |
| Year of diagnosis: 2014 vs. 2012 | - | 0.68 | 0.40 | 1.14 | 0.141 | - | 0.63 | 0.50 | 0.79 | <.0001 |
| Tumor size: >5–≤10 cm vs. ≤5 cm | - | 0.91 | 0.59 | 1.40 | 0.674 | - | 0.85 | 0.59 | 1.20 | 0.352 |
| Tumor size: >10 cm vs. ≤5 cm | - | 0.83 | 0.50 | 1.40 | 0.490 | - | 0.67 | 0.46 | 0.97 | 0.034 |
| Primary site: gastric vs. non-gastric | - | 1.02 | 0.69 | 1.49 | 0.936 | - | 1.47 | 1.11 | 1.94 | 0.007 |
| Imatinib starting dosage: ≥400 mg vs. <400 mg | - | 0.84 | 0.45 | 1.56 | 0.584 | - | 0.88 | 0.66 | 1.15 | 0.346 |
| **Advanced/metastatic setting** | | | | | | | | | | |
|  | Japan | | | | | Taiwan | | | | |
|  | Unadjusted HR | Adjusted HR | 95% CI | | P-value | Unadjusted HR | Adjusted HR | 95% CI | | P-value |
|  |  |  | LL | UL |  |  |  | LL | UL |  |
| Age: ≥75 vs <75 years | 1.06 | 1.17 | 0.77 | 1.79 | 0.458 | 1.62 | 1.58 | 1.22 | 2.05 | 0.001 |
| Sex: female vs. male | - | 1.49 | 1.07 | 2.08 | 0.018 | - | 0.84 | 0.68 | 1.06 | 0.154 |
| Year of diagnosis: 2013 vs. 2012 | - | 1.79 | 1.13 | 2.85 | 0.014 | - | 1.12 | 0.85 | 1.46 | 0.421 |
| Year of diagnosis: 2014 vs. 2012 | - | 2.00 | 1.24 | 3.21 | 0.004 | - | 1.10 | 0.83 | 1.46 | 0.505 |
| Tumor size: >5–≤10 cm vs. ≤5 cm | - | 0.87 | 0.54 | 1.38 | 0.545 | - | 1.37 | 0.86 | 2.18 | 0.186 |
| Tumor size: >10 cm vs. ≤5 cm | - | 0.97 | 0.61 | 1.55 | 0.893 | - | 1.18 | 0.75 | 1.86 | 0.465 |
| Distant metastasis: yes vs. no | - | 0.83 | 0.57 | 1.20 | 0.311 | - | 0.79 | 0.59 | 1.05 | 0.098 |
| Primary site: gastric vs. non-gastric | - | 1.12 | 0.80 | 1.57 | 0.498 | - | 1.28 | 0.95 | 1.71 | 0.102 |
| Imatinib starting dosage: ≥400 mg vs. <400 mg | - | 1.16 | 0.70 | 1.93 | 0.569 | - | 1.09 | 0.79 | 1.50 | 0.621 |

HR, hazard ratio; CI, confidence interval; LL, lower limit, UL, upper limit

Missing values in the tumor size and distant metastasis were analyzed in the model as indicator variables.

# Supplementary Table 5. Multivariable Cox regression analysis of the time to stop imatinib with additional variables (Taiwan only)

| **Adjuvant setting** | | | | |
| --- | --- | --- | --- | --- |
|  | Adjusted HR | 95% CI | | P-value |
|  |  | LL | UL |  |
| Age: ≥75 vs. <75 years | 1.48 | 1.14 | 1.92 | 0.003 |
| Sex: female vs. male | 1.01 | 0.83 | 1.22 | 0.946 |
| Year of diagnosis: 2013 vs. 2012 | 0.92 | 0.71 | 1.19 | 0.525 |
| Year of diagnosis: 2014 vs. 2012 | 0.67 | 0.52 | 0.87 | 0.002 |
| Tumor size: >5–≤10 cm vs. ≤5 cm | 0.80 | 0.56 | 1.14 | 0.212 |
| Tumor size: >10 cm vs. ≤5 cm | 0.61 | 0.41 | 0.89 | 0.010 |
| Mitotic count: high vs. low | 0.83 | 0.64 | 1.07 | 0.144 |
| Primary site: gastric vs. non-gastric | 1.33 | 1.00 | 1.77 | 0.053 |
| Imatinib starting dosage: ≥400 mg vs. <400 mg | 0.92 | 0.69 | 1.21 | 0.535 |
| Charlson comorbidity index: ≥2 vs. 0, 1 | 1.50 | 1.18 | 1.90 | 0.001 |
| **Advanced/metastatic setting** | | | | |
|  | Adjusted HR | 95% CI | | P-value |
|  |  | LL | UL |  |
| Age: ≥75 vs. <75 years | 1.59 | 1.23 | 2.07 | 0.001 |
| Sex: female vs. male | 0.83 | 0.66 | 1.04 | 0.111 |
| Diagnosed year: 2013 vs. 2012 | 1.15 | 0.87 | 1.53 | 0.326 |
| Diagnosed year: 2014 vs. 2012 | 1.15 | 0.86 | 1.55 | 0.355 |
| Tumor size: >5–≤10 cm vs. ≤5 cm | 1.36 | 0.85 | 2.17 | 0.197 |
| Tumor size: >10 cm vs. ≤5 cm | 1.19 | 0.75 | 1.87 | 0.461 |
| Mitotic count: high vs. low | 1.23 | 0.85 | 1.77 | 0.277 |
| Distant metastasis: yes vs. no | 0.77 | 0.58 | 1.02 | 0.073 |
| Primary site: gastric vs. non-gastric | 1.27 | 0.94 | 1.70 | 0.116 |
| Imatinib starting dosage: ≥400 mg vs. <400 mg | 1.06 | 0.77 | 1.47 | 0.713 |
| Charlson comorbidity index: ≥2 vs. 0, 1 | 0.95 | 0.74 | 1.21 | 0.671 |

HR, hazard ratio; CI, confidence interval; LL, lower limit, UL, upper limit

Supplementary Table 6. Multivariable Cox regression analysis for overall survival (Taiwan only)

| Adjuvant setting | | | | |
| --- | --- | --- | --- | --- |
|  | adjusted HR | 95% CI | | p value |
|  |  | LL | UL |  |
| age >=75 vs < 75 | 3.78 | 2.23 | 6.41 | <.0001 |
| female vs male | 0.67 | 0.39 | 1.12 | 0.126 |
| diagnosed year: 2013 vs 2012 | 1.62 | 0.79 | 3.32 | 0.193 |
| diagnosed year: 2014 vs 2012 | 1.63 | 0.82 | 3.25 | 0.165 |
| Tumor size: >5 ~ ≤ 10 vs ≤5 | 1.86 | 0.54 | 6.46 | 0.328 |
| Tumor size: > 10 vs ≤5 | 1.76 | 0.49 | 6.32 | 0.389 |
| mitotic count: high vs low | 1.03 | 0.53 | 1.99 | 0.926 |
| primary site: gastric vs non-gastric | 1.58 | 0.71 | 3.53 | 0.266 |
| Imatinib starting dosage: >=400 mg vs <400 mg | 1.18 | 0.59 | 2.33 | 0.644 |
| Charlson comorbidity index: >=2 vs 0, 1 | 3.29 | 1.92 | 5.62 | <.0001 |
| Advanced/metastatic setting | | | | |
|  | adjusted HR | 95% CI | | p value |
|  |  | LL | UL |  |
| age >=75 vs < 75 | 2.53 | 1.85 | 3.45 | <.0001 |
| female vs male | 0.71 | 0.53 | 0.94 | 0.017 |
| diagnosed year: 2013 vs 2012 | 1.23 | 0.86 | 1.74 | 0.255 |
| diagnosed year: 2014 vs 2012 | 1.52 | 1.06 | 2.17 | 0.022 |
| Tumor size: >5 ~ ≤ 10 vs ≤5 | 1.24 | 0.63 | 2.45 | 0.529 |
| Tumor size: > 10 vs ≤5 | 1.63 | 0.86 | 3.07 | 0.134 |
| mitotic count: high vs low | 1.05 | 0.64 | 1.72 | 0.838 |
| primary site: gastric vs non-gastric | 1.01 | 0.73 | 1.41 | 0.939 |
| Imatinib starting dosage: >=400 mg vs <400 mg | 0.89 | 0.61 | 1.31 | 0.562 |
| Charlson comorbidity index: >=2 vs 0, 1 | 1.05 | 0.78 | 1.42 | 0.753 |

# Supplementary Document 1. GIST registry in the Taiwan Cancer Registry

**Gastrointestinal stromal tumors (GISTs) in the Taiwan Cancer Registry (TCR)**

The malignancy of gastric and non-gastric GISTs was determined according to the World Health Organization prognostic group and American Joint Committee on Cancer (AJCC), 7th edition. When the prognostic groups of gastric GISTs are classified as “1,” “2,” “3a,” or “4”; or “1” for non-gastric GISTs, these groups are considered as benign tumors, and hence, they are not reported to the TCR. The others are considered malignant tumors and are reported to the TCR. Malignant GIST tumors are reported according to the International Classification of Diseases for Oncology Third Edition histological code 8936/3. The details are as follows:

| AJCC Stage | Tumor size  (cm) | Mitotic rate (per 50 HPFs) | Revised NIH risk (Joensuu, 2008) § | Prognostic group | Reported or not to the TCR? |
| --- | --- | --- | --- | --- | --- |
| Gastric GISTs | | | | | |
| Stage IA | ≤2 cm | Low (≤5) | Very low | 1 | No |
| Stage IA | >2 cm–≤5 cm | Low (≤5) | Low | 2 | No |
| Stage IB | >5 cm–≤10 cm | Low (≤5) | Intermediate | 3a | No |
| Stage II | >10 cm | Low (≤5) | High | 3b | Yes |
| Stage II | ≤2 cm | High (>5) | Intermediate or High | 4 | No |
| Stage II | >2 cm–≤5 cm | High (>5) | Intermediate or High | 5 | Yes |
| Stage IIIA | >5 cm–≤10 cm | High (>5) | High | 6a | Yes |
| Stage IIIB | >10 cm | High (>5) | High | 6b | Yes |
| Non-gastric GISTs | | | | | |
| Stage I | ≤2 cm | Low (≤5) | Very low | 1 | No |
| Stage I | >2 cm–≤5 cm | Low (≤5) | Low | 2 | Yes |
| Stage II | >5 cm–≤10 cm | Low (≤5) | High | 3a | Yes |
| Stage IIIA | >10 cm | Low (≤5) | High | 3b | Yes |
| Stage IIIA | ≤2 cm | High (>5) | Intermediate or High | 4 | Yes |
| Stage IIIB | >2 cm–≤5 cm | High (>5) | High | 5 | Yes |
| Stage IIIB | >5 cm–≤10 cm | High (>5) | High | 6a | Yes |
| Stage IIIB | >10 cm | High (> 5) | High | 6b | Yes |

HPF, high power field; NIH, National Institutes of Health

§ Joensuu, H. Risk stratification of patients diagnosed with gastrointestinal stromal tumor. *Hum. Pathol.* **39**(10),1411-1419 (2008).

# Supplementary Document 2. Definitions of the setting and prescription patterns

**Definitions of the settings**

We grouped the patients with gastrointestinal stromal tumors (GISTs) into three settings because each scenario has different recommended treatment courses in the clinical guidelines. The timeframe of the settings is further shown in Supplementary Figure 1. We defined the settings as follows:

1. Adjuvant setting

The patients who received adjuvant imatinib therapy were defined as those with no distant metastasis who underwent surgery and started with imatinib therapy <168 days (24 weeks) after surgery.

1. Advanced/metastatic disease setting

The patients with advanced/metastatic GISTs were defined as those with distant metastasis, who used two or more types of tyrosine kinase inhibitor (TKI) drugs, those who received TKI therapy without undergoing surgery, those who started postoperative TKI therapy >24 weeks after surgery, those who received neoadjuvant TKI therapy within the past year before surgery, and postoperative TKI therapy started >24 weeks after surgery, or those who underwent surgery >48 weeks after imatinib initiation.

1. Neoadjuvant setting

Those who received neoadjuvant therapy were categorized in this group. The patients who received neoadjuvant therapy were excluded since neoadjuvant treatment for GISTs was not a standard therapy at this point.

**Definitions of imatinib prescription patterns**

After identifying their pathological conditions, we divided the patients into three groups: (1) continued group, (2) discontinued group, and (3) switched group. We defined these groups because imatinib is the first-line treatment for patients, and if the patients experienced imatinib intolerance or disease progression, they will either discontinue treatment or switch to the second-line treatment, sunitinib. An attempt to increase the imatinib dosage was not considered because the insurance reimbursement policy does not approve it in either Japan or Taiwan (see Supplementary Document 4 for details).

Operationally, we defined the continued group as follows: those who received imatinib without changing to other drugs until December 31, 2018. Interruption of imatinib treatments for <8 weeks or initiation of imatinib treatment from 24 weeks after surgery were allowed. The discontinued group was defined as those who started imatinib at first but discontinued for some reason without switching to sunitinib or regorafenib. The switched group was defined as those who started with imatinib but switched to sunitinib or regorafenib. The timeframes of the three patterns are further shown in Supplementary Figures 2 and 3.

# Supplementary Document 3. Detailed steps of the extraction of the Japanese and Taiwanese data

**Japanese study cohort**

The Hospital-Based Cancer Registry (HBCR) and Diagnosis Procedure Combination (DPC) databases were used to identify eligible patients with gastrointestinal stromal tumors (GISTs). It is mandatory to report both benign and malignant GISTs to the HBCR. Therefore, the patients with GISTs in the Japanese cohort were identified by morphological codes of “89361” and “89363” from 2012 to 2014.

Patient extraction steps

First, we identified patients with a morphological code of GIST (International Classification of Diseases for Oncology Third Edition morphological codes: 89361 and 89363) from the HBCR between January 1, 2012, and December 31, 2014. Then, we only extracted the records that had the “class of case (coc)” variable in the HBCR coded as 2, 3, or 4. We mainly extracted the data of patients with “coc” ≥2 or 3, indicating that they received initial cancer treatment within the same hospital. We also extracted the data of patients with a “coc” = 4, revealing the patients who returned to a facility after receiving initial treatment at a different facility.

Second, we linked our cohort to the DPC database. Since we had two different DPC data sources with different identification numbers, we matched patients using their facility identification number, date of birth, and procedure and medication codes. After matching, we identified the target medications (imatinib, sunitinib, and regorafenib) within the cohort. Patients who started tyrosine kinase inhibitor (TKI) therapy with sunitinib or regorafenib were excluded. The timeframe of our study was until December 31, 2018.

**Taiwanese study cohort**

The Taiwan Cancer Registry (TCR) and National Health Insurance (NHI) databases were used to identify eligible patients with GISTs in this study. In the TCR, only malignant GISTs are reported. Therefore, the patients with GISTs in the Taiwan cohort were identified using a morphological code of “89363” from 2012 to 2014.

Patient extraction steps

First, we identified patients with a histology code of GIST (ICD-O-3 morphological code: 89363) from the Taiwan Cancer Registry Long Form and Short Form (Long Form variable: HIST and Short Form variable: HISTBEH) between January 1, 2012, and December 31, 2014.

Second, when patients had multiple records, we only extracted the records wherein the “class” variable was coded as 1 or 2 because it suggests that the patients received all cancer treatments within the same hospital (variable: CLASS). We used the pathological stage (TCR long form, variable: PSTAGE) to confirm the patients’ stage. If the pathological stage was not available, we used the clinical stage instead (TCR long form, variable: CSTAGE). Finally, we used the Cancer Registry Annual Report File to confirm the diagnosed date (variable: DIAG_DT).

The next step was to link our cohort to the NHI database. We used the Details of Ambulatory Care Orders (variable: DRUG_NO) and Details of Inpatient Orders (variable: ORDER_CODE) to identify the TKI drugs. Three TKIs were analyzed in our study: imatinib, sunitinib, and regorafenib. The date of treatment initiation was obtained from the Ambulatory Care Expenditures by Visits (variable: FUNC_DATE) and Inpatient Expenditures by Admissions (variable: IN_DATE). Patients who started TKI therapy with sunitinib or regorafenib were excluded. The timeframe of our study was until December 31, 2018.

# Supplementary Document 4. Reimbursement for the GIST-targeted therapies in the Taiwan National Health Insurance Program

**The Taiwan National Health Insurance (NHI) Reimbursement for the** **GIST-targeted therapy between 2012 and 2018**

Imatinib:

1. For unresectable or metastatic gastrointestinal stromal tumors (GISTs), no pre-authorization review was needed.
2. For adjuvant therapy:
3. Tumor size ≥6 cm: The NHI reimbursed 1 year after approval of the pre-authorization review (February 2011–August 2013).
4. If one of the following criteria was met, the NHI reimbursed 3 years after the pre-authorization review (September 2013):

- Tumor size >10 cm
- Mitotic rate >10/50 high power field (HPF)
- Tumor size >5 cm and mitotic rate >5/50 HPF
- Tumor rupture

Sunitinib:

1. Limited to treat GISTs after failure of imatinib therapy due to resistance or intolerance.
2. A switch to imatinib after the failure of sunitinib due to resistance or intolerance was not permitted.
3. For the pre-authorization review, the medical service organization provided medical records and proof (evidence) of intolerance or ineffectiveness of imatinib.

Regorafenib:

1. Patients with locally advanced, unresectable, or metastatic GISTs previously treated with imatinib and sunitinib.
2. For the pre-authorization review, the treatment duration for each application was limited to 3 months, and the image data were required for the review every 3 months.

**Pre-authorization review:** According to the “National Health Insurance Pharmaceutical Benefit and Reimbursement Scheme,” for certain special materials and drugs that are at high risk, expensive, or prone to be used indiscriminately, the insurer shall conduct a prior review. The medical service organization should submit applications including medical records, medical plan, laboratory data, pathology or cytology data, and image data to the National Health Insurance Administration (NHIA). The NHIA will appoint medical experts in relevant specialties to review the application materials, and complete the verification within 2 weeks after receiving the application documents.

# Supplementary Figure 1. Diagram of the patient conditions

[Note: OP, surgery; TKI, tyrosine kinase inhibitors]


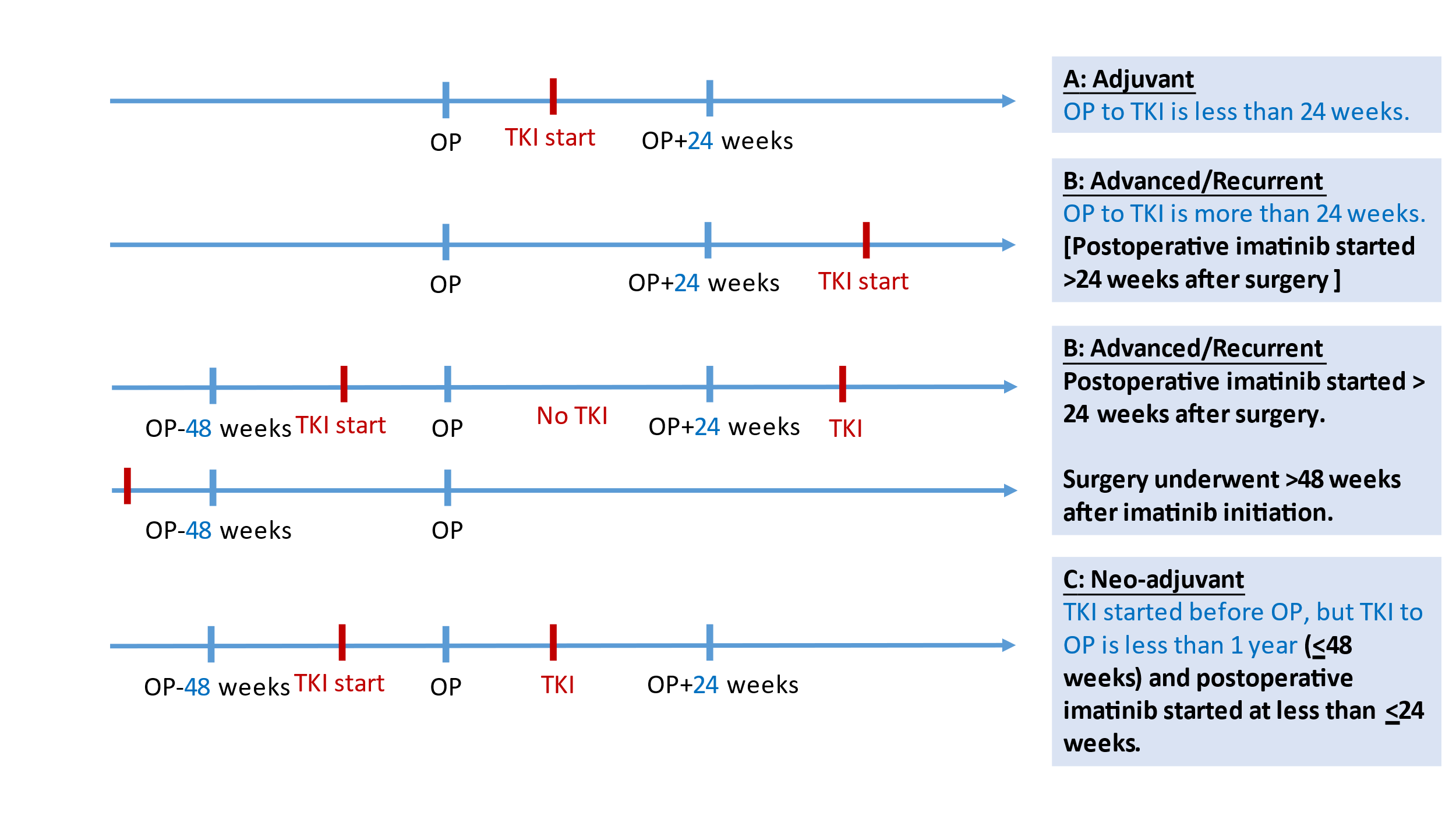


# Supplementary Figure 2. Illustration of the imatinib treatment pattern groups in the adjuvant setting


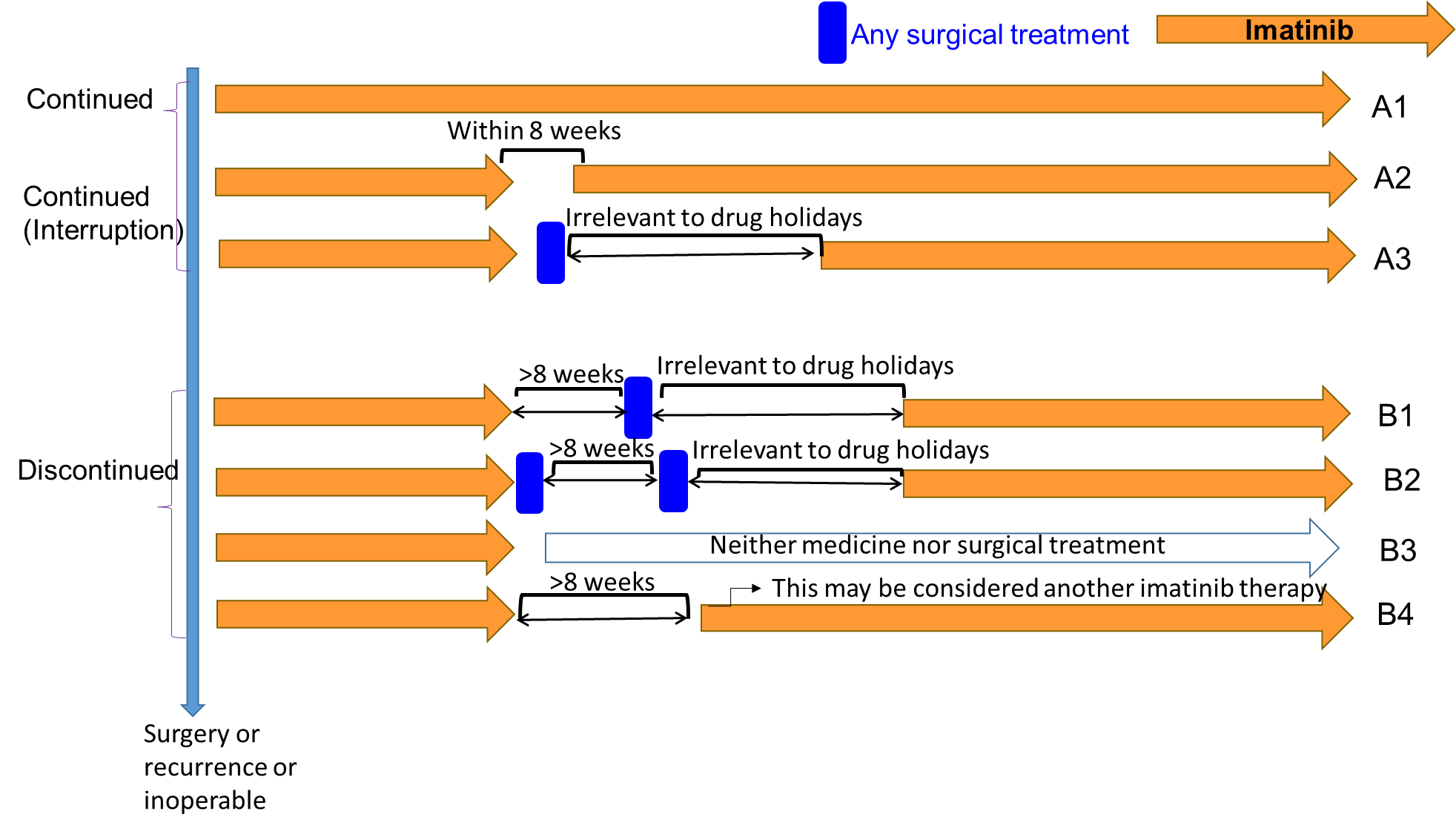


# Supplementary Figure 3. Illustration of the imatinib treatment pattern groups in the advanced/metastatic setting


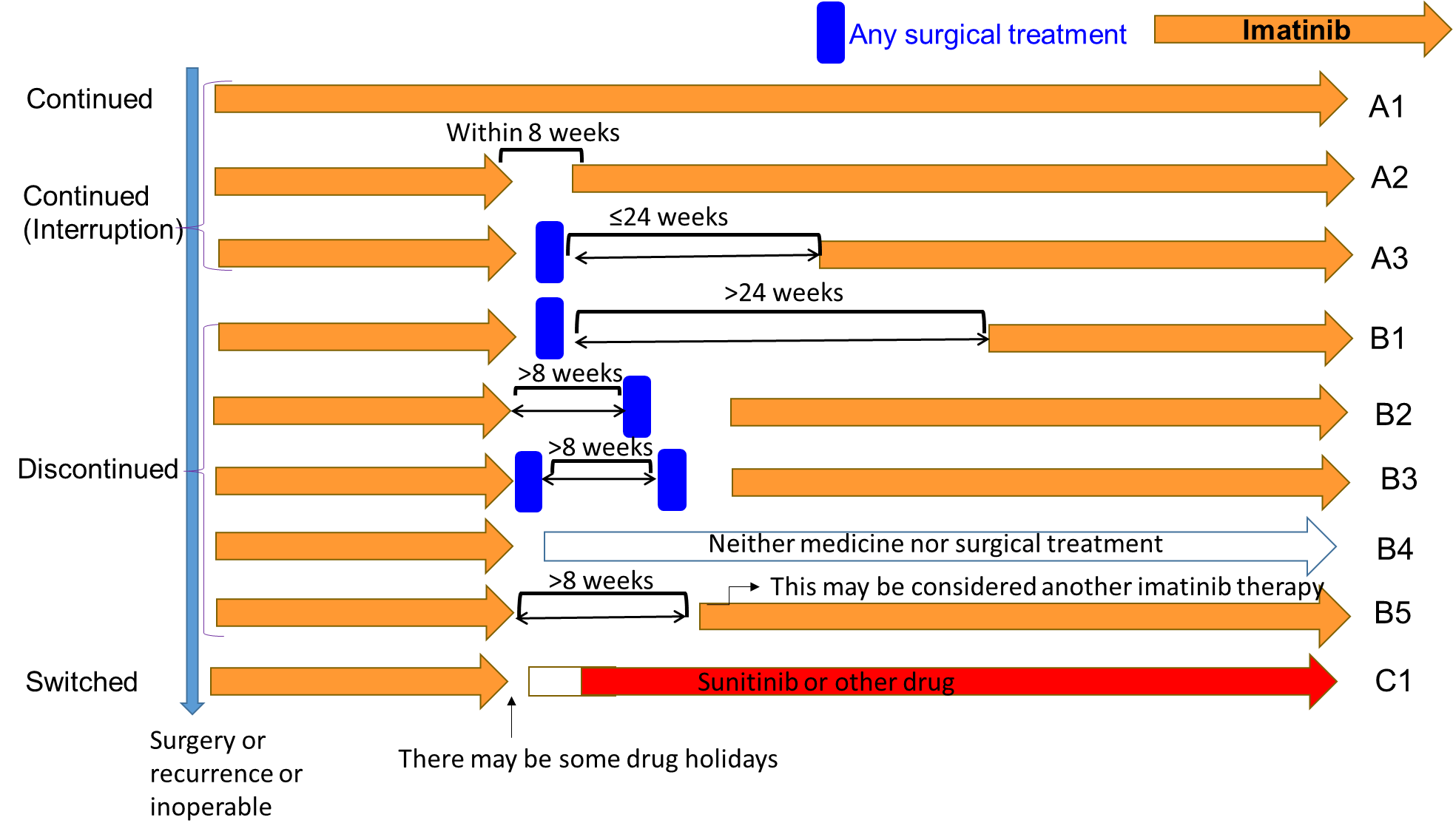


#
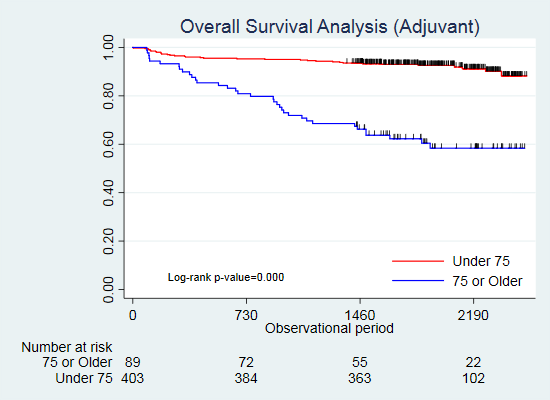
Supplementary Figure 4. Kaplan Meier analysis for overall survival (Taiwan only)

1965 days

Median days = 815

Overall survival analysis (advanced/metastatic)

Overall survival analysis (adjuvant)


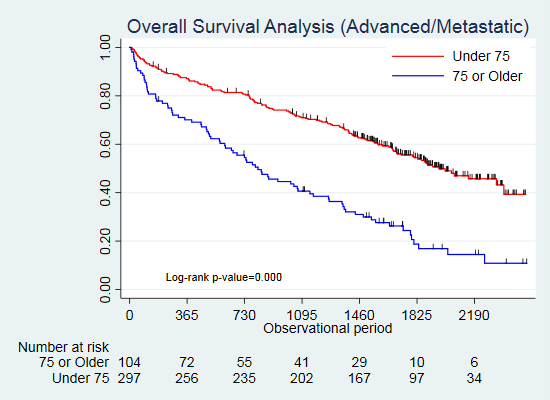
(a) Adjuvant setting (b) Advanced/metastatic setting
